# A Resilience-Based Nursing Intervention Framework for Internet Gaming Disorder in Saudi University Health Settings

**DOI:** 10.3390/healthcare14162599

**Published:** 2026-08-18

**Authors:** Faihan F. Alshaibany, Bader M. Almutairy, Alya Alghamdi, Waleed M. Alshehri, Majed M. Aljabri, Abdullah Alharbi, Bandar S. Alharbi, Abdulaziz M. Alodhailah

**Affiliations:** 1 Department of Nursing Administration and Education, College of Nursing, King Saud University, Riyadh 11451, Saudi Arabia; 2 Community and Psychiatric Mental Health Nursing Department, College of Nursing, King Saud University, Riyadh 11451, Saudi Arabia; 3 Department of Medical-Surgical Nursing, College of Nursing, King Saud University, Riyadh 11451, Saudi Arabia

**Keywords:** internet gaming disorder, psychological resilience, impulsivity, behavior change wheel, COM-B model, cultural adaptation, Saudi Arabia, complex intervention development, stepped care, Islamic psychology

## Abstract

**Background/Objectives:** Internet Gaming Disorder (IGD) is included in ICD-11 and poses a substantial risk to Saudi university students, yet no structured nursing intervention framework exists within Saudi higher education health services. This paper presents the Resilience-Based Nursing Intervention Framework (RBNIF), a three-tiered, theory-driven nursing architecture translating evidence on impulsivity, resilience, and IGD into practice specifications for this setting. **Methods:** Using secondary analysis of a dataset of 207 gaming students from four King Saud University colleges, framework development followed a four-phase Medical Research Council (MRC) process: theoretical identification (I-PACE, Dual Process Theory, COM-B), evidence synthesis, Behavior Change Wheel/Intervention Mapping, and cultural adaptation via Bernal et al.’s Ecological Validity Framework. **Results:** The sample (58.9% male; 20–25 years, 61.4%; bachelor’s level, 73.9%) yielded three tiers differentiated by a dual-domain screening rule (elevated impulsivity and/or low resilience, rather than a single combined score): Tier 1 (universal psychoeducation); Tier 2 (six-session group program, S-UPPS-P ≥ 52 and/or CD-RISC-10 ≤ 20; 63 of 207 participants [30.4%]); and Tier 3 (10-session RIRDH clinical program, IGDS9-SF ≥ 36, met by 38 of 207 participants [18.4%]). Fifty-two participants (25.1%) met the elevated-impulsivity criterion, and 51 (24.6%) met the low-resilience criterion. Each tier specifies nursing competencies and behavior change techniques, with cultural adaptation integrating Islamic values concepts (sabr, mizan, tawakkul), family protocols, and single-sex delivery. **Conclusions:** To our knowledge, the RBNIF is the first theory-driven, culturally adapted nursing intervention architecture for IGD in Saudi university health services; a focused, non-systematic search of PubMed, Scopus, and CINAHL identified no directly comparable published framework. It is pre-implementation and requires feasibility testing, pilot evaluation, and a cluster-randomized controlled trial (c-RCT).

## 1. Introduction

Internet Gaming Disorder (IGD) was included in the eleventh revision of the International Classification of Diseases (ICD-11), adopted by the World Health Assembly in 2019 and brought into effect in 2022, and has since been documented across university student populations internationally, with meta-analytic estimates of gaming disorder prevalence in the low single digits globally [1]. Within the Gulf Cooperation Council and neighboring Arab university populations, however, reported gaming disorder/addiction risk has been markedly higher, ranging from approximately 6% to 19%, with associations reported for depression, anxiety, and academic performance indicators such as GPA [2,3,4,5,6]. Saudi-specific studies among university and medical students have reported prevalence estimates in a broadly similar range [2,3,4], while adolescent samples in the Kingdom show comparable patterns [7]; epidemiological work among Egyptian university students has documented similar concerns regionally [8]. In the sample underlying the present framework, 18.4% of 207 gaming students at four King Saud University colleges met probable IGD caseness (IGDS9-SF ≥ 36) [9], which is consistent with this elevated regional pattern rather than with the lower prevalence typically reported in general-population meta-analyses [1], a difference attributable in part to the restriction of the denominator to students who already game rather than to all students.

Impulsivity and psychological resilience are among the most consistently reported risk and protective factors for IGD. Systematic reviews and meta-analyses link elevated impulsivity to gaming disorder severity across populations [10,11,12], while resilience has been shown to buffer this association. In the dataset underlying the present framework, moderation analysis demonstrated that psychological resilience attenuates the association between impulsivity and IGD severity, and structural equation modeling indicated that resilience contributes to the impulsivity–IGD relationship through both direct protective and indirect mediating pathways [9]. Sex-stratified analyses further suggested that the impulsivity–IGD association may be stronger among male students, whereas the protective influence of resilience appeared consistent across sex [9]. Full measurement and psychometric detail for these analyses, including the derivation of separate percentile-based impulsivity and resilience screening thresholds, is reported in the study [9] and is not repeated here; the present paper addresses a distinct question, namely how these empirical parameters can be translated into a nursing intervention architecture.

Published gaming disorder interventions are predominantly East Asian in origin, with a smaller body of European work [13,14,15]; these programs were developed for specialist clinic or hospital-based delivery contexts that differ substantially from Saudi university health service structures, staffing norms, and cultural expectations. No published framework has been designed for nurse-led delivery within Saudi or wider Gulf university health services. What the existing literature does not provide, and what the present paper directly addresses, is the translational architecture connecting these evidence-based targets to nursing practice; specifying which nursing role delivers which intervention, to which students, at what intensity, using which specific BCTs, and how that delivery should be systematically adapted for the Saudi sociocultural context. Saudi university students operate within a sociocultural system shaped by Islamic values, gender-differentiated norms, and extended family structures; an intervention that ignores this risks poor uptake and cultural mismatch, so cultural adaptation here draws explicitly on Islamic frameworks of health and self-regulation [16] and on gender-sensitive delivery.

This paper presents the Resilience-Based Nursing Intervention Framework (RBNIF), a three-tiered nursing intervention architecture for IGD prevention and treatment in Saudi university health settings.

## 2. Materials and Methods

### 2.1. Study Design and Data Source

This study presents a secondary analysis and theory-driven framework development study using an existing dataset rather than a new primary empirical investigation. The dataset comprised 207 Saudi university students who reported current video gaming engagement, recruited through stratified cluster convenience sampling from four colleges at King Saud University. Data collection was conducted for the companion empirical study reported by Alshaibany et al. (2026) [9] under the King Saud University Institutional Review Board (IRB), with written informed consent obtained from all participants. The companion study reports the measurement properties and structural relationships among impulsivity, resilience, and IGD severity using structural equation modeling; although both papers use the same underlying dataset, they address distinct research questions: psychometric structure and pathway testing in the companion study versus dual-domain risk-threshold derivation and intervention framework development here and provide complementary rather than duplicative contributions.

The present study used the existing dataset to establish separate percentile-based risk thresholds for impulsivity and resilience, applied jointly as a dual-domain screening rule rather than combined into a single composite score for the Short UPPS-P Impulsive Behavior Scale (S-UPPS-P), Connor–Davidson Resilience Scale-10 (CD-RISC-10), and Internet Gaming Disorder Scale–Short Form (IGDS9-SF). These empirical parameters informed the development of the theory-driven, culturally adapted three-tiered nursing intervention framework described in subsequent sections. The derivation of these thresholds, including the ROC/AUC analysis (AUC = 0.80) and full psychometric detail on the measurement models for these instruments, is reported in the companion study [9]; it is not re-derived here.

### 2.2. Reporting Guidelines

As this study describes the development of a complex intervention framework based on secondary analysis of an existing dataset, rather than a new observational study or qualitative inquiry, reporting was guided by the Medical Research Council (MRC) framework for developing and evaluating complex interventions [17]. and the Template for Intervention Description and Replication (TIDieR) checklist [18]. Reporting was additionally benchmarked against the GUIDED checklist for intervention development studies [19]. Completed GUIDED and TIDieR checklists are provided as Supplementary Files S1 and S2, respectively. The primary empirical study from which the dataset was derived has reported its observational methods and findings separately [9]. Ethics approval for the original data collection (King Saud University IRB, reference 26-0350) is on file. The study characteristics and correlations are presented in Table 1. The original participant consent explicitly extends to secondary use of the dataset for framework development. Therefore, the present study focuses on framework development, intervention specification, and theoretical translation rather than repeating the reporting elements of the parent study.

The framework development process was assessed against the TIDieR checklist. Items related to intervention rationale, materials, procedures, delivery approach, provider characteristics, setting, timing, and dosage are described in the framework development sections. Adaptation procedures are presented in the cultural adaptation subsection. Items related to implementation modifications, fidelity assessment, and actual delivery cannot yet be reported because the RBNIF remains in the pre-implementation phase and will be evaluated during future feasibility testing.

Framework development proceeded through four iterative phases following the MRC guidance [20]. Phase 1 (Theory identification) drew on the I-PACE model [21], Dual Process Theory [22], and the Stress-and-Coping model [23] to generate a causal model specifying the psychological mechanisms through which the RBNIF should produce change. Phase 2 (Evidence synthesis) integrated findings from available empirical data on IGD in Saudi university students and from systematic reviews of gaming disorder interventions [13] and resilience promotion programs in university settings [24]. Phase 3 (Modeling) applied the Behavior Change Wheel and Intervention Mapping protocol to translate theoretical targets and evidence-based content into intervention functions, delivery modes, and session-level behavior change techniques. Phase 4 (Cultural adaptation) applied Bernal et al.’s [25] Ecological Validity Framework for cultural adaptation. Cultural adaptation was informed by published qualitative literature on mental health help-seeking and gaming behaviors among Saudi and Gulf Arab youth, and by expert consultation with Saudi mental health nursing professionals during framework drafting. No primary qualitative data collection was conducted in this framework development phase; planned feasibility testing (Phase 1) will incorporate direct stakeholder involvement with nursing staff and student participants.

Two distinct CD-RISC-10 values appear in this study and its companion [9] and serve different purposes. The value of 32.7 is the threshold above which the companion moderation analysis found resilience to exert a statistically significant protective effect on the impulsivity–IGD association; it describes a continuous moderation effect rather than a screening cut-point. The value of 20 is the 25th percentile of CD-RISC-10 scores in the present sample and is used, in combination with the S-UPPS-P 75th percentile (≥52), as the Tier 2 screening criterion. A student scoring between 20 and 32.7 on the CD-RISC-10 is not automatically assigned to Tier 2 on resilience grounds alone: Tier 2 eligibility requires CD-RISC-10 ≤ 20 and/or S-UPPS-P ≥ 52 as specified in Table 2, so a student in the 20–32.7 range is eligible only if the S-UPPS-P criterion is also met; otherwise, that student remains at Tier 1.

### 2.3. COM-B Analysis

COM-B analysis of the target behaviors (reduced gaming disorder severity, increased resilience use in response to gaming urges, impulsivity management) identified three primary behavioral barriers. Capability barriers included insufficient self-awareness of impulsive gaming patterns, limited knowledge of the IGD diagnostic framework, and underdeveloped cognitive reappraisal and urge-surfing skills. Opportunity barriers included limited access to mental health support within university settings, absence of structured psychoeducational materials in Arabic addressing gaming disorder, and social norms that normalize excessive gaming in male peer groups. Motivation barriers included ambivalence about gaming as a problem behavior (reinforced by positive gaming experiences), low perceived vulnerability to gaming disorder, and help-seeking stigma within the Saudi student context.

COM-B analysis directed the selection of intervention functions: education (to address capability), enablement (to address opportunity), persuasion and incentivization (to address motivation), and environmental restructuring (to address opportunity). These functions map onto specific behavior change techniques (BCTs) at the session level, detailed in Table 3.

## 3. Results

### 3.1. Sample Characteristics

Table 1 presents the demographic characteristics, gaming patterns, descriptive statistics, and correlations among the primary study variables. The sample consisted of 207 students, including 122 males (58.9%) and 85 females (41.1%). The modal age group was 20–25 years (61.4%, n = 127), and most participants were enrolled at the bachelor’s level (73.9%, n = 153). Daily Gaming Hours demonstrated a right-skewed distribution, with 34.3% reporting 1–3 h of daily gaming and 17.4% reporting more than 6 h. Descriptive statistics and intercorrelations indicated that IGD severity was positively associated with impulsivity and gaming hours and negatively associated with resilience.

Table 1C presents the sample-specific thresholds used for the Tier 2 and Tier 3 screening classifications together with the observed classification frequencies. The elevated-impulsivity threshold (S-UPPS-P ≥ 52) corresponds to the sample 75th percentile; 52 of 207 participants (25.1%) met this criterion. The low-resilience threshold (CD-RISC-10 ≤ 20) corresponds to the sample 25th percentile; 51 of 207 participants (24.6%) met this criterion. The Tier 2 classification criterion combines these two domains: 63 of 207 participants (30.4%) met S-UPPS-P ≥ 52 and/or CD-RISC-10 ≤ 20 while not meeting the IGD caseness threshold (IGDS9-SF < 36). The Tier 3 classification (probable IGD) includes 38 of 207 participants (18.4%) meeting the IGDS9-SF ≥ 36 threshold. Table 1B provides the reported descriptive statistics and supplementary distributional characteristics for IGDS9-SF, confirming the observed distribution aligns with the reported caseness proportion.

### 3.2. The Resilience-Based Nursing Intervention Framework

**Figure 1:**
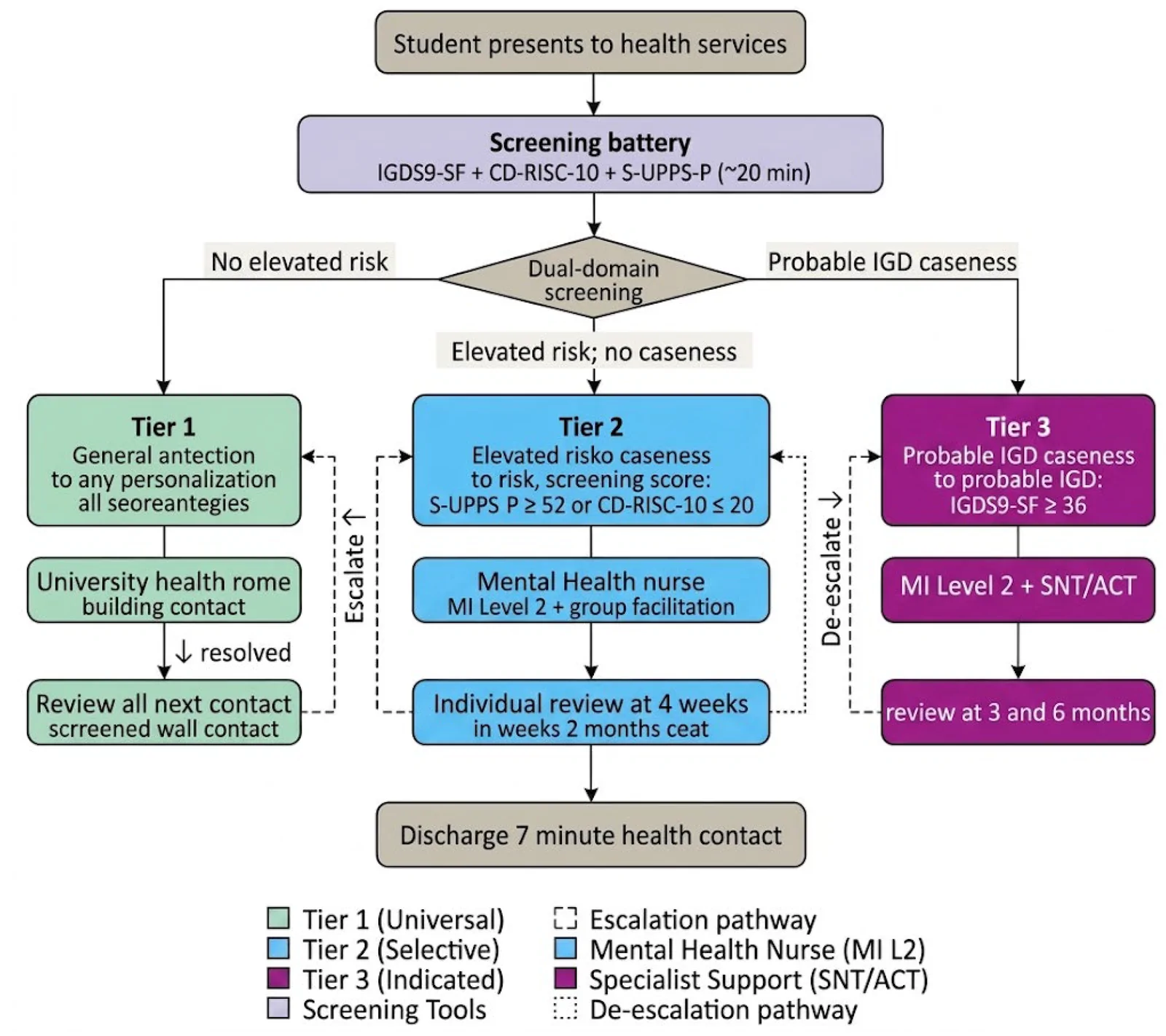
Visual schematic of the RBNIF three-tier architecture, illustrating patient entry, dual-domain screening decision points, tier allocation pathways, escalation and de-escalation routes, and follow-up schedule.

Figure 1 presents a visual schematic of the RBNIF’s three-tier architecture, illustrating patient flow, screening decision points, and referral pathways between tiers. The RBNIF is organized into three service tiers differentiated by a dual-domain impulsivity/resilience screening rule and IGD severity threshold. Table 2 presents the full tier architecture.

Tier 1 is a universal prevention tier targeting all students who present to university health services and disclose regular gaming engagement, without restriction by risk score.

Tier 2 is a selective early intervention tier targeting students with elevated risk profiles: S-UPPS-P score at or above the 75th percentile (≥52) and/or CD-RISC-10 score at or below the 25th percentile (≤20), but not meeting conservative IGD caseness (IGDS9-SF < 36). In the present sample, 63 of 207 students (30.4%) met this criterion.

Tier 3 is an indicated clinical intervention tier for students meeting conservative IGD caseness (IGDS9-SF ≥ 36). In the present sample, 38 of 207 students (18.4%) met this criterion.

Tier eligibility is determined at the point of university health service contact through a brief digital screening battery (IGDS9-SF + CD-RISC-10 + S-UPPS-P, total approximately 20 min), automatically scoring and flagging students who meet the impulsivity and/or resilience screening criteria.

#### 3.2.1. Tier 1: Universal Prevention

Tier 1 provides universal gaming wellness content to all students who disclose gaming engagement during routine health service contact. The core deliverable is a structured 20 min psychoeducational contact covering: (a) normative information about gaming behaviors and the distinction between recreational and disordered gaming; (b) psychoeducation about how impulsivity and low resilience interact to escalate gaming disorder risk, using accessible explanations grounded in the dual-process framework; (c) a brief self-monitoring challenge (seven days of self-reported gaming hours and emotional state associated with gaming sessions); and (d) signposting to the university’s digital wellness resources, including a self-monitoring app.

Tier 1 also functions as the gateway to the screening battery: students who complete the psychoeducational contact are offered the IGDS9-SF + CD-RISC-10 + S-UPPS-P screening battery, and results are automatically scored and used to determine Tier 2 or Tier 3 eligibility. The universal framing, available to all gaming students rather than specifically targeting students suspected of having a problem, reduces stigma-based barriers to uptake and provides a non-stigmatizing context for introducing the concept of gaming disorder risk.

#### 3.2.2. Tier 2: Selective Early Intervention

Tier 2 is delivered as a structured six-session group program for students identified as high-risk under the impulsivity/resilience screening rule but not yet screening positive for probable IGD. Sessions are 90 min each and are facilitated by a mental health nurse in collaboration with a counseling psychology student services staff member. Groups are capped at eight participants and are run in single-sex format (separately for male and female students) to align with Saudi cultural norms and to enable frank discussion of gaming behaviors in gender-relevant social contexts.

Session content follows a CBT-informed resilience and risk awareness curriculum: Session 1 (Understanding Gaming Disorder Risk), Session 2 (My Impulsivity Profile, introducing the UPPS-P model in accessible language), Session 3 (Understanding Resilience, personal resilience mapping using the CD-RISC-10 items as a self-exploration tool), Session 4 (Urge Recognition and Surfing), Session 5 (Building Alternative Coping Resources), and Session 6 (Personalized Gaming Wellness Plan and Forward Commitment). An individual follow-up at four weeks assesses whether gaming hours have changed and whether further support is warranted. Students who develop caseness (IGDS9-SF ≥ 36) during Tier 2 follow-up are escalated to Tier 3.

#### 3.2.3. Tier 3: The RIRDH Program

Tier 3 delivers the Resilience and Impulsivity Regulation for Digital Health (RIRDH) program to students screening positive for probable IGD (i.e., meeting the conservative IGDS9-SF caseness threshold), pending clinical assessment. RIRDH is a 10-session structured intervention integrating CBT, Acceptance and Commitment Therapy (ACT), and motivational interviewing elements within an explicitly resilience-building framework. Table 3 provides the full session outline with content and behavior change techniques for each session.

### 3.3. Nursing Competency Requirements

Effective RBNIF delivery requires a differentiated nursing competency profile across the three tiers. Table 4 details competency requirements. Tier 1 can be delivered by any university health nurse following a half-day induction covering gaming disorder psychoeducation, brief screening tool administration, and referral pathway orientation. Tier 2 requires a mental health nursing qualification (registered mental health nurse or equivalent) with additional training in group facilitation, motivational interviewing, and CBT-informed psychoeducation delivery. Tier 3 (RIRDH) requires a qualified mental health nurse with verified competence in CBT, ACT-based urge surfing, motivational interviewing, and relapse prevention. Clinical supervision (minimum monthly individual supervision plus quarterly group case formulation, per Table 4) is mandatory for all Tier 3 practitioners throughout the delivery period.

### 3.4. Cultural Adaptation for the Saudi University Context

#### 3.4.1. Islamic Values Integration

The most consequential cultural adaptation involves integrating Islamic values frameworks as genuine therapeutic resources rather than surface-level additions. This approach is consistent with the growing literature on culturally adapted mental health interventions for Muslim populations, which identifies Islamic psychological concepts as clinically relevant constructs that map onto evidence-based behavioral techniques [16,26,27]. Islamic psychology offers several concepts directly relevant to gaming disorder management. The concept of sabr (patient endurance under adversity), elaborated in classical Islamic ethics as an active regulatory capacity rather than passive resignation [26], urges tolerance and distress tolerance skills; students may find it easier to engage with urge surfing when it is framed as an expression of sabr rather than a purely clinical technique. The concept of mizan (balance and moderation across all life domains), which has theological grounding in Qur’anic injunctions on balance and equity [27], provides a culturally resonant framework for discussing healthy gaming limits without pathologizing gaming itself, consistent with an addiction-sensitive approach that avoids pathologizing behavior while acknowledging harm thresholds. The concept of tawakkul (active trust in divine providence combined with personal agency and effort) can be mobilized as a resilience resource within the CD-RISC-10’s spiritual dimension [28], acknowledging that for many Saudi students, religious commitment and identity function as active sources of psychological resilience rather than merely a source of coping in the background [29].

Session 6 (Building Resilience Resources) and Session 7 (Healthy Coping Repertoire) explicitly incorporate these frameworks. The facilitator guide for these sessions includes worked examples of how sabr and mizan might be applied to specific gaming disorder scenarios, and culturally adapted worksheets allow students to map their religious and community resources alongside the standard resilience domains assessed by the CD-RISC-10.

#### 3.4.2. Family Involvement

Family relationships may be an important source of support for some Saudi students and may also shape opportunities to set gaming boundaries. Accordingly, the family communication component is optional, student-led, and subject to assessment of family safety and relationship dynamics. Session 8 (Gaming Rules and Boundaries) includes an optional family communication module in which students develop and rehearse a family conversation about gaming limits. Family information sessions, delivered separately from the student program, are offered to parents who request them. These sessions provide psychoeducation on gaming disorder and explain the RBNIF process without disclosing confidential session content. Family involvement is framed as optional and student-led, preserving autonomy while acknowledging the relational context within which gaming behavior occurs.

#### 3.4.3. Gender Sensitivity

All Tier 2 and Tier 3 groups are delivered in a single-sex format. Male-only groups are facilitated by male nursing staff; female-only groups by female nursing staff. This arrangement aligns with Saudi cultural norms around gender mixing and facilitates more open discussion of gaming-related social experiences within gender-relevant peer contexts. Facilitators are trained to remain attentive to sex- and context-related differences in gaming experiences reported in the companion study [9], but core Tier 2 and Tier 3 content is consistent across male and female groups; the previously proposed differential emphasis (impulsivity-management content for men, social/environmental content for women) is not retained, since the underlying finding indicates a stronger statistical association in men rather than a demonstrated difference in treatment need. Any sex-specific adaptation will be co-designed with students and evaluated during feasibility testing.

### 3.5. Implementation and Evaluation Plan

Table 5 presents the phased implementation and evaluation plan. Following framework finalization, Phase 1 (Feasibility Testing, months 1–6) involves manual piloting of the screening battery and Tier 1 contact at one university site, with user experience feedback collected from nursing staff and student participants. For the purposes of the Phase 1 success criterion in Table 5, an adverse event is defined as any clinically significant deterioration in student wellbeing, safeguarding concern, or study withdrawal due to distress that a reviewing nurse attributes at least in part to the screening or Tier 1 contact. Phase 2 (Pilot Implementation, months 7–18) delivers all three tiers at two contrasting university sites (one large public university, one smaller private institution), with mixed-methods process evaluation documenting fidelity, reach, dose, and preliminary outcomes. Phase 3 (Effectiveness Evaluation, months 19–42) is a cluster-randomized controlled trial at a minimum of six sites, with IGD caseness at 6 months as the primary outcome and IGDS9-SF severity, CD-RISC-10, and S-UPPS-P scores as secondary outcomes.

## 4. Discussion

Several features distinguish the RBNIF from existing gaming disorder intervention frameworks. To our knowledge, no published framework has been systematically designed for nurse-led delivery in Arabic within an Islamic cultural context and targeted specifically at university health services (databases searched: PubMed, Scopus, and CINAHL; gaming disorder/IGD combined with intervention, framework, nursing, and Saudi/GCC terms, through mid-2026). Existing programs such as the Korean Jump Up Internet Rescue School [15] were developed for specialist clinic or inpatient settings with psychologist-led delivery, and systematic reviews of gaming disorder interventions [14] likewise describe predominantly specialist or hospital-based delivery. Saudi IGD research spans diverse subpopulations, from general university and medical students [2,3,4] to clinical subgroups such as students with autism spectrum disorder [30], underscoring the need for population-specific thresholds rather than thresholds imported from heterogeneous East Asian samples. Grounding the framework in empirical data from the same population, instruments, and theoretical constructs gives the RBNIF an internal consistency that generic adaptation of external programs could not achieve.

### 4.1. Rationale for the Tiered Architecture

The choice of a tiered service architecture reflects both the epidemiological findings and pragmatic health system considerations. With 18.4% of student gamers in this sample meeting the predefined probable-IGD criterion, a resource-intensive individual therapy model may not be feasible for the full population at risk within typical university health service budgets. The tiered model reserves intensive resources (Tier 3/RIRDH) for students classified as probable IGD while using cost-efficient universal screening (Tier 1) and structured group programming (Tier 2) for the larger subclinical-risk population, consistent with the stepped-care approach endorsed by the WHO Mental Health Gap Action Programme [31] and Saudi Arabia’s Vision 2030 health system efficiency goals [32,33].

The dual-domain Tier 2 classification criterion (S-UPPS-P ≥ 52 and/or CD-RISC-10 ≤ 20, excluding participants meeting the Tier 3 criterion) identifies a substantial minority of screened gaming students for Tier 2 early intervention. In the present sample, 63 of 207 participants (30.4%) met the Tier 2 classification criterion. These confirmed participant-level frequencies provide an empirical basis for initial service-planning estimates.

For operational planning purposes, and assuming hypothetically that the observed 30.4% Tier 2 proportion were replicated in a larger university population, a university enrolling 1000 gaming students would be expected to have approximately 304 Tier 2-eligible students. With Tier 2 groups capped at eight participants per group, serving all Tier 2-eligible students would require 38 groups (⌈304/8⌉ = 38). Each group requires nine hours of direct group delivery time across six 90 min sessions, yielding approximately 342 h of direct group delivery time for the Tier 2 caseload, excluding preparation, documentation, supervision, and individual follow-up.

Similarly, assuming the observed Tier 3 proportion of 18.4% were replicated, approximately 184 of 1000 gaming students would meet the Tier 3 classification criterion. If these students received the 10-session RIRDH program, and each session required 60–90 min of direct delivery per student, the corresponding direct delivery requirement would be approximately 1840–2760 h per 1000 gaming students if all sessions were delivered individually. Actual resource requirements would be lower if sessions 3–9 were delivered in small-group formats, depending on group size and delivery configuration.

These staffing and service-planning projections are grounded in the confirmed classification frequencies from the present sample but remain hypothetical population-level estimates. They should be refined using prospective data on screening uptake, clinical assessment confirmation, attendance patterns, group size, delivery format, and completion rates during Phase 1 feasibility testing and Phase 2 pilot implementation. Accordingly, the estimates provide preliminary workforce-planning guidance for university service-capacity discussions rather than fixed resource requirements.

### 4.2. Theoretical Basis and Behavior Change Content

The COM-B analysis provided important insights that shaped intervention content. The identification of motivation barriers, particularly ambivalence about gaming as a problem and low perceived vulnerability, directed the selection of motivational interviewing as a core Tier 3 technique rather than immediate skills training: students who do not perceive a problem are unlikely to engage with impulsivity or cognitive-restructuring content until this ambivalence is resolved [10,11,12]. The RIRDH’s first two sessions accordingly prioritize engagement, functional analysis, and decisional balance over prescriptive skills training; Session 1 introduces goal-setting only as an initial orientation exercise conducted collaboratively within a motivational interviewing frame, rather than as a fixed behavioral prescription, and more directive change-focused content follows from Session 3 onward. This sequencing is evidence-based [34] and culturally appropriate in a context where help-seeking for behavioral health issues carries stigma. The behavior change techniques specified in the RIRDH session outline (Table 3) are drawn from the Behavior Change Wheel taxonomy [35] and from the general CBT and ACT evidence base for gaming disorder treatment, predominantly developed and tested in East Asian settings [13]; direct transferability to Saudi students cannot be assumed, and cultural adaptation is a necessary but not sufficient condition for equivalence. Whether the specific BCTs selected produce the expected changes in gaming behavior within Saudi students’ lives remains an empirical question that only feasibility testing and pilot evaluation can answer.

### 4.3. Cultural Adaptation for the Saudi Context

The cultural adaptation process identified family as both a potential resource and a potential barrier. Saudi students’ extended family networks may reinforce gaming-limiting behaviors when family members are informed and supportive, or may inadvertently enable gaming disorder by providing unrestricted device access or social reinforcement for gaming achievement; the RBNIF’s optional, student-led family communication module navigates this dual role without compromising student autonomy. The Islamic values integration (sabr, mizan, tawakkul) is not intended as an instrumental persuasion tactic but reflects genuine equivalents between the framework’s therapeutic constructs and Islamic psychological traditions that many students already draw on [16,26,27,28,29], consistent with broader evidence linking Islamic religiosity to student mental wellbeing [36]. Surfacing these equivalences allows students to approach the intervention as consistent with, rather than alien to, their cultural identity, an approach more robust than simple translation and more respectful than cultural override.

### 4.4. Clinical and Policy Implications

Three implications follow, framed as options for feasibility testing rather than recommendations for routine adoption given the framework’s pre-implementation status. First, universities considering IGD support services may pilot the RBNIF’s screening approach (IGDS9-SF combined with the CD-RISC-10/S-UPPS-P dual-domain rule) alongside existing student mental health pathways, with prospective evaluation of the thresholds before wider use. Second, where feasibility testing supports it, budgets could plan for a gaming-disorder-related nursing role at the mental health nurse level, using the Tier 2/3 competency profile as a starting specification; Section 4.1 nurse-time estimates are planning projections meant to inform, not substitute for, that evaluation. Third, pilot sites could be co-located with existing student mental health services for psychiatric liaison on IGD-comorbid presentations, with Arabic-language RIRDH materials developed for eventual GCC scale-up. These are health system transformation goals rather than purely clinical ones: Saudi university health services are predominantly staffed by general health nurses with limited specialist mental health capacity [37], so meeting the Tier 2/3 competency requirements implies either recruiting specialist nurses or investing in upskilling, requiring advocacy from university leadership, professional bodies, and the Saudi Commission for Health Specialties.

### 4.5. Strengths and Limitations

The principal strength of the RBNIF is its population-specific development, with screening thresholds, intervention targets, and session content informed by empirical data from the intended population. The framework also specifies tier-differentiated nursing competencies and session-level behavior-change strategies to support future implementation. The confirmed Tier 2 (30.4%) and Tier 3 (18.4%) frequencies provide an empirical basis for preliminary service planning; however, both the thresholds and frequencies were derived from a single cross-sectional sample of 207 students from four King Saud University colleges and should not be interpreted as population prevalence estimates.

Several limitations warrant consideration. The RBNIF has not yet been tested in practice, and nurse-time estimates are planning projections rather than observed requirements. The percentile-based thresholds were derived and evaluated within the same dataset, creating potential overfitting; independent cross-validation and prospective validation in other Saudi and Gulf universities are therefore required before wider implementation. The thresholds are screening classifications rather than newly established diagnostic criteria and should be refined using clinical, behavioral, and multi-institutional data. Generalizability is also limited by the single-institution, cross-sectional design and exclusive reliance on self-report measures.

Implementation may be constrained by limited availability of CBT/ACT-trained mental health nurses and the absence of a formal workforce capacity analysis. Family involvement requires assessment of family availability and suitability, with individual alternatives and safeguarding procedures where necessary. Consistent with the GUIDED checklist (Supplementary File S1), the development process also lacked stakeholder-facing prototype testing and systematic documentation of discarded components; participatory engagement with students and other stakeholders is planned for Phase 1.

Finally, the proposed Phase 3 cluster-randomized controlled trial will require substantial institutional and inter-university collaboration. Phase 2 pilot data can inform interim service-planning decisions. Future research should prioritize participatory design, independent and multi-institutional validation, workforce capacity analysis, evaluation of Islamic-values components, digital delivery adaptations, and implementation-science approaches such as CFIR [38].

## 5. Conclusions

The RBNIF provides a structured, theory-informed, and culturally adapted nursing framework for IGD in Saudi university health services. Its three-tier architecture integrates dual-domain impulsivity–resilience screening, proportionate intervention intensity, COM-B-informed behavior-change strategies, and tier-specific nursing competencies.

To our knowledge, no directly comparable nursing framework for IGD in Saudi university health services has been published. However, this conclusion is based on a focused, non-systematic literature search and should therefore be interpreted cautiously.

The RBNIF is pre-implementation and requires feasibility testing and pilot evaluation across contrasting Saudi university sites before wider application, followed by the proposed cluster-randomized controlled trial to establish its effectiveness and implementation feasibility.

## Figures and Tables

**Table 1:** Demographic characteristics, gaming patterns, descriptive statistics, and correlations among study variables (N = 207).

**(A) Sample Characteristics**			**n**		**%**	
**Sex**						
Male			122		58.9	
Female			85		41.1	
**Age Band**						
<20 years			28		13.5	
20–25 years			127		61.4	
26–30 years			39		18.8	
>30 years			13		6.3	
**Academic Level**						
Diploma			18		8.7	
Bachelor’s			153		73.9	
Master’s			28		13.5	
Doctorate			8		3.9	
**Daily Gaming Hours**						
<1 h			41		19.8	
1–3 h			71		34.3	
4–6 h			59		28.5	
>6 h			36		17.4	
**(B) Descriptive statistics, correlations, and IGDS9-SF distributional characteristics.**						
**Variable**	**M**	**SD**	**1**	**2**	**3**	**4**
1. IGD Severity (IGDS9-SF)	22.4	7.8	—			
2. Impulsivity (S-UPPS-P)	46.3	10.2	0.47 ***	—		
3. Resilience (CD-RISC-10)	25.6	7.1	−0.33 ***	−0.31 ***	—	
4. Daily Gaming Hours ^a^	—	—	0.39 ***	0.22 **	−0.18 **	—
**IGDS9-SF** **Distributional** **Characteristics**						
**Statistic**					**Value**	
Mean					22.4	
SD					7.8	
Median					21.0	
Q1 (25th percentile)					17.0	
Q3 (75th percentile)					26.0	
IQR					9.0	
Skewness					0.72	
Minimum					9	
Maximum					45	
**(C) Empirically Derived Percentile-Based Classification Thresholds and Confirmed Frequencies (N = 207)**						
**Classification Category**	**Threshold Definition**			**Frequency in This Sample**		
Elevated Impulsivity	S-UPPS-P ≥ 52 (75th percentile of the sample)			52/207 (25.1%)		
Low Resilience	CD-RISC-10 ≤ 20 (25th percentile of the sample)			51/207 (24.6%)		
Tier 2 Classification	S-UPPS-P ≥ 52 and/or CD-RISC-10 ≤ 20; IGDS9-SF < 36			63/207 (30.4%)		
Tier 3 Classification (Probable IGD)	IGDS9-SF ≥ 36			38/207 (18.4%)		

Notes: Panel B: ** *p* < 0.01; *** *p* < 0.001. ^a^ Daily Gaming Hours was recorded as an ordered categorical variable; associations involving this variable were therefore estimated using Spearman’s rank correlation rather than Pearson’s r. Note on distributional characteristics: The IGDS9-SF exhibits a positively skewed distribution (skewness = 0.72), with median (21.0) lower than mean (22.4), consistent with a right-skewed pattern. This right-skewed distribution explains the observed caseness rate of 18.4% at the score of 36, which exceeds the approximately 4% expected under a normal distribution with the same mean and standard deviation. Under normality, a score of 36 would correspond to z = 1.74 and thus 4.1% of the distribution; the heavier upper tail of the actual right-skewed distribution accounts for the higher observed proportion. Panel C: Classification frequencies are confirmed participant-level values derived from the complete dataset of 207 students. The thresholds were empirically derived from sample-specific percentile cut-points and have not been externally validated. Accordingly, these categories should be interpreted as screening classifications rather than clinical diagnoses. External validation in independent and, preferably, multi-institutional samples is recommended before broader application of the classification framework. Tier 3 eligibility takes precedence over Tier 2 eligibility when assigning participants to intervention tiers.

**Table 2:** Resilience-Based Nursing Intervention Framework (RBNIF): three-tier architecture.

Tier	Eligibility	Intervention	Nursing Role	Frequency
Tier 1	All gaming students presenting to health services	Gaming wellness psychoeducation; CD-RISC-10 and S-UPPS-P brief screening; self-monitoring app	University health nurse; student health promotion officer	1–2 contacts per semester
Tier 2	S-UPPS-P ≥ 52 and/or CD-RISC-10 ≤ 20; IGDS9-SF < 36 (63/207 [30.4%] in this sample)	Six-session resilience and risk psychoeducation group; gaming-behavior self-regulation skills	Mental health nurse; student counseling service co-facilitator	Six 90 min group sessions; one individual follow-up at 4 weeks
Tier 3	IGDS9-SF ≥ 36 (probable IGD classification; 38/207 [18.4%] in this sample)	10-session RIRDH program (individual and group components); psychiatric liaison if comorbidity is identified	Mental health/psychiatric liaison nurse; CBT-trained counselor	10 sessions over 12 weeks; follow-up at 3 and 6 months

Notes: Thresholds were empirically derived from the 207-student sample recruited from four colleges at King Saud University using sample-specific percentile-based cut-points (see Section 2.1 and Section 3.1). The reported classification frequencies (Tier 2: 30.4%; Tier 3: 18.4%) are confirmed participant-level values. These thresholds are sample-derived and have not yet been externally validated in independent or multi-institutional samples. Accordingly, the classification framework should be considered preliminary and should undergo external validation before wider application. Tier 3 eligibility takes precedence over Tier 2 eligibility when assigning participants to intervention tiers.

**Table 3:** RIRDH program session outline.

Session	Title	Core Content	Behavior Change Techniques
1	Assessment and Engagement	IGD psychoeducation; gaming history timeline; motivational interviewing; goal-setting	Information provision; goal setting; motivational interviewing techniques
2	My Gaming Profile	Functional analysis of gaming behavior; reinforcement schedule identification; pros/cons decisional balance	Self-monitoring; functional analysis; decisional balance worksheet
3	Understanding Impulsivity	UPPS-P model introduction; personal impulsivity profile; urge recognition and the urge cycle	Self-monitoring; psychoeducation on urge cycle; cue identification
4	Surfing the Urge	Urge surfing technique (mindful urge observation); distress tolerance skills; cue-exposure without response	Urge surfing; mindfulness practice; graded exposure to gaming cues
5	Thinking Traps and Cognitive Reappraisal	Common cognitive distortions in gaming (‘just one more game’; sunk cost fallacy); thought records; cognitive reappraisal training	Cognitive restructuring; thought records; reappraisal practice
6	Building Resilience Resources	CD-RISC-10 profile exploration; personal resilience strengths mapping; social, spiritual, and family resources identification	Strengths-based assessment; values clarification; social resource mapping
7	Healthy Coping Repertoire	Alternative coping strategy development; behavioral activation; activity scheduling using Islamic framework of balanced living (mizan)	Behavioral activation; problem-solving; scheduling alternative reinforcers
8	Gaming Rules and Boundaries	Gaming time contracts; stimulus control; environmental modifications; family involvement in boundary-setting	Stimulus control; commitment devices; family-inclusive boundary-setting
9	Academic and Social Wellbeing	Integration of gaming management with academic demands; social reconnection; identifying non-gaming achievement domains	Social support mobilization; values-based goal review; academic engagement strategies
10	Review and Forward Planning	IGDS9-SF and CD-RISC-10 reassessment; individual outcome review; relapse prevention planning; follow-up scheduling	Relapse prevention planning; follow-up commitment; progress review and self-reinforcement

Notes: Sessions 1–2 individual (60 min); sessions 3–9 group (90 min, 4–6 participants); session 10 individual (60 min).

**Table 4:** RBNIF nursing competency requirements by tier.

Tier	Required Qualification	Required Training	Supervision
Tier 1	Registered Nurse (any field)	Half-day RBNIF induction; gaming disorder psychoeducation certificate	Annual competency review; Tier 2/3 nurse available for consultation
Tier 2	Registered Mental Health Nurse or equivalent	Motivational interviewing Level 2; group facilitation training; 3-day RBNIF Tier 2 certification	Bi-monthly individual clinical supervision; peer supervision group
Tier 3	Registered Mental Health Nurse with CBT/ACT competence certification	CBT Level 2; ACT urge surfing; relapse prevention certification; RIRDH program facilitator training (3 days)	Monthly individual clinical supervision (mandatory); case formulation group quarterly; psychiatric liaison access

Notes: All tiers require cultural competency training in Saudi mental health help-seeking norms.

**Table 5:** RBNIF Implementation and Evaluation Plan.

Phase	Timeline	Activity	Evaluation Method	Success Criteria
1: Feasibility	Months 1–6	Manual screening battery administration at 1 site; Tier 1 delivery; nursing staff training; user experience data collection	Semi-structured interviews; screening feasibility audit; staff competency assessment	≥80% screening completion; staff satisfaction ≥ 4/5; <10% adverse events
2: Pilot	Months 7–18	All 3 tiers at 2 sites; process evaluation; fidelity monitoring; preliminary pre-post outcome data	Fidelity checklist; reach and dose monitoring; pre-post IGD severity, CD-RISC-10, S-UPPS-P	Tier 3 IGDS9-SF reduction ≥ 5 points; CD-RISC-10 improvement ≥ 4 points; 80% session attendance
3: RCT	Months 19–42	Cluster-RCT at ≥6 sites; 1:1 allocation intervention vs. usual care; 6-month primary outcome; 12-month follow-up	IGDS9-SF caseness at 6 months; secondary outcomes CD-RISC-10, S-UPPS-P, academic functioning, wellbeing	Significant reduction in caseness (OR ≤ 0.60); CONSORT-compliant reporting

## Data Availability

The data presented in this study are available on request from the corresponding author due to ethical restrictions related to participant confidentiality and the potential risk of re-identification within a single-university student sample; no qualitative interview data were collected for this study.

## Supplementary Materials

The following supporting information can be downloaded at: https://www.mdpi.com/article/10.3390/healthcare14162599/s1, Supplementary File S1: Completed GUIDED (GUidance for the rEporting of intervention Development) checklist for the RBNIF framework development process. Supplementary File S2: Completed COREQ (Consolidated Criteria for Reporting Qualitative Research) checklist for the RBNIF pre-implementation intervention specification.

## Abbreviations

The following abbreviations are used in this manuscript. Abbreviations are defined at first use in the text:

RIRDH	Resilience and Impulsivity Regulation for Digital Health
CBT	Cognitive Behavioral Therapy
ACT	Acceptance and Commitment Therapy
IGD	Internet Gaming Disorder
RBNIF	Resilience-Based Nursing Intervention Framework
MRC	Medical Research Council
COM-B	Capability, Opportunity, Motivation—Behavior model
BCW	Behavior Change Wheel
BCT	Behavior Change Technique
MI	Motivational Interviewing
I-PACE	Interaction of Person-Affect-Cognition-Execution model
IGDS9-SF	Internet Gaming Disorder Scale–9 Short-Form
CD-RISC-10	Connor-Davidson Resilience Scale, 10-item version
S-UPPS-P	Short UPPS-P Impulsive Behavior Scale
AUC	Area Under the (ROC) Curve
WHO	World Health Organization
GCC	Gulf Cooperation Council
IRB	Institutional Review Board
TIDieR	Template for Intervention Description and Replication
c-RCT	Cluster-Randomized Controlled Trial
ROC	Receiver Operating Characteristic
DSM-5	Diagnostic and Statistical Manual of Mental Disorders, 5th Edition

## References

[1] Stevens M.W.R., Dorstyn D., Delfabbro P.H., King D.L. (2021). Global Prevalence of Gaming Disorder: A Systematic Review and Meta-Analysis. Aust. N. Z. J. Psychiatry.

[2] Khrad H., Marhoomi A., Alkhiri A., Alshomrani A., Bajabir D., Mosli M. (2022). Prevalence of Internet Gaming Disorder among Saudi Arabian University Students: Relationship with Psychological Distress. Heliyon.

[3] Alsunni A.A., Latif R. (2022). Internet Gaming Disorder and Its Correlates among University Students, Saudi Arabia. J. Fam. Community Med..

[4] Al Asqah M.I., Al Orainey A.I., Shukr M.A., Al Oraini H.M., Al Turki Y.A. (2020). The Prevalence of Internet Gaming Disorder among Medical Students at King Saud University, Riyadh, Saudi Arabia: A Cross-Sectional Study. Saudi Med. J..

[5] Al Azizi K., Al Omari O., Abu Sharour L., Damra J.K., Al Aamri M., Al Rahbi W., Al Abri B., Al Yahyaei A., Abdalrahim A., Albashtawy M. (2026). Prevalence of Gaming Addiction and Its Associations with Stress, Anxiety, Depression and Self-Esteem among University Students: A Cross-Sectional Study. Clin. Child Psychol. Psychiatry.

[6] Almutairi T.A., Almutairi K.S., Ragab K.M., Nourelden A.Z., Assar A., Matar S., Rashid H.H., Elsayed M., Fathallah A.H., Spitzer M. (2023). Prevalence of Internet Gaming Disorder and Its Association with Psychiatric Comorbidities among a Sample of Adults in Three Arab Countries. Middle East Curr. Psychiatry.

[7] Alghamdi M.H., Alghamdi M.M. (2023). Prevalence of Internet Gaming Disorder Among Intermediate and High School Students in Albaha, Saudi Arabia: A Cross-Sectional Study. Cureus.

[8] Shouman A., Elez W.A., Ibrahim I.M.A., Elwasify M. (2023). Internet Gaming Disorder and Psychological Well-Being among University Students in Egypt. BMC Psychol..

[9] Alshaibany F.F., Aljabri M.M., Alharbi A.M., Alharbi B.S., Alghamdi A., Almutairy B.M., Alshehri W.M., Alyahya N.M., Alodhailah A.M. (2026). A Structural Equation Model of Impulsivity, Psychological Resilience, and Internet Gaming Disorder: Testing Direct and Indirect Pathways Among Saudi University Students. Healthcare.

[10] Nuske J., Nuske L., Stevens M.W.R., Billieux J., Delfabbro P.H., Hides L., Johnson D., King D.L. (2026). The Association between Gaming Disorder and Impulsivity: A Systematic Review and Meta-Analysis. Aust. N. Z. J. Psychiatry.

[11] Aulia K., Utami M.S.S. (2025). Cognitive Behavioral Therapy (CBT) Approach In Adolescents with Online Game Addiction. Eduvest-J. Univers. Stud..

[12] Andrade A.L.M., Lobato F.B.H., Stange N., Scatena A., Oliveira W.A.D., Kim H.S., Lopes F.M. (2024). The Association between Gaming Disorder and Impulsivity: A Systematic Review. Estud. Psicol. Camp..

[13] King D.L., Delfabbro P.H., Wu A.M.S., Doh Y.Y., Kuss D.J., Pallesen S., Mentzoni R., Carragher N., Sakuma H. (2017). Treatment of Internet Gaming Disorder: An International Systematic Review and CONSORT Evaluation. Clin. Psychol. Rev..

[14] Chen Y., Lu J., Wang L., Gao X. (2023). Effective Interventions for Gaming Disorder: A Systematic Review of Randomized Control Trials. Front. Psychiatry.

[15] Koo C., Wati Y., Lee C.C., Oh H.Y. (2011). Internet-Addicted Kids and South Korean Government Efforts: Boot-Camp Case. Cyberpsychol. Behav. Soc. Netw..

[16] Hamdan A. (2008). Cognitive Restructuring: An Islamic Perspective. J. Muslim Ment. Health.

[17] Craig P., Dieppe P., Macintyre S., Michie S., Nazareth I., Petticrew M. (2008). Developing and Evaluating Complex Interventions: The New Medical Research Council Guidance. BMJ.

[18] Hoffmann T.C., Glasziou P.P., Boutron I., Milne R., Perera R., Moher D., Altman D.G., Barbour V., Macdonald H., Johnston M. (2014). Better Reporting of Interventions: Template for Intervention Description and Replication (TIDieR) Checklist and Guide. BMJ.

[19] Duncan E., O’Cathain A., Rousseau N., Croot L., Sworn K., Turner K.M., Yardley L., Hoddinott P. (2020). Guidance for reporting intervention development studies in health research (GUIDED): An evidence-based consensus study. BMJ Open.

[20] Moore G.F., Audrey S., Barker M., Bond L., Bonell C., Hardeman W., Moore L., O’Cathain A., Tinati T., Wight D. (2015). Process Evaluation of Complex Interventions: Medical Research Council Guidance. BMJ.

[21] Brand M., Wegmann E., Stark R., Müller A., Wölfling K., Robbins T.W., Potenza M.N. (2019). The Interaction of Person-Affect-Cognition-Execution (I-PACE) Model for Addictive Behaviors: Update, Generalization to Addictive Behaviors beyond Internet-Use Disorders, and Specification of the Process Character of Addictive Behaviors. Neurosci. Biobehav. Rev..

[22] Evans J.S.B.T. (2008). Dual-Processing Accounts of Reasoning, Judgment, and Social Cognition. Annu. Rev. Psychol..

[23] Lazarus R.S., Folkman S. (1984). Stress, Appraisal, and Coping.

[24] Abulfaraj G.G., Upsher R., Zavos H.M.S., Dommett E.J. (2024). The Impact of Resilience Interventions on University Students’ Mental Health and Well-Being: A Systematic Review. Educ. Sci..

[25] Bernal G., Bonilla J., Bellido C. (1995). Ecological Validity and Cultural Sensitivity for Outcome Research: Issues for the Cultural Adaptation and Development of Psychosocial Treatments with Hispanics. J. Abnorm. Child Psychol..

[26] Aqtam I. (2026). The Psychology of Sabr: Toward a Conceptual Framework of Resilience and Coping for Palestinian Muslims Facing Collective Trauma. npj Ment. Health Res..

[27] Shaker A.F. (2015). Man, Existence and the Life Balance (Mīzān) in Islamic Philosophy. J. Islam. Stud..

[28] Gondal M.U., Adil A., Niazi S. (2021). Tawakkul Mediates between Religious Orientation and Depression among Muslims. Clin. Couns. Psychol. Rev..

[29] Koenig H.G., Al Shohaib S. (2014). Health and Well-Being in Islamic Societies: Background, Research, and Applications.

[30] Eltantawy M.M., Almutairi M. (2026). Internet Gaming Disorder among Individuals with Autism Spectrum Disorder in the Kingdom of Saudi Arabia: A Comparative Study. Front. Public Health.

[31] World Health Organization (2016). mhGAP Intervention Guide for Mental, Neurological and Substance Use Disorders in Non-Specialized Health Settings: Mental Health Gap Action Programme (Version 2.0).

[32] Saudi Vision 2030 (2016). Vision 2030: Kingdom of Saudi Arabia.

[33] Kingdom of Saudi Arabia (2021). Vision 2030: National Transformation Program—Health Sector.

[34] Miller W.R., Rollnick S. (2013). Motivational Interviewing: Helping People Change.

[35] Michie S., Van Stralen M.M., West R. (2011). The Behaviour Change Wheel: A New Method for Characterising and Designing Behaviour Change Interventions. Implement. Sci..

[36] Hapsari P., Darodjat (2025). Correlation Between Islamic Religiosity and Mental Well-Being in Students in the Perspective of Achieving Sustainable Development Goals (SDGs). Profetika J. Studi Islam.

[37] Qureshi N.A., AlHabeeb A.A., Koenig H. (2013). Mental Health System in Saudi Arabia: An Overview. Neuropsychiatr. Dis. Treat..

[38] Damschroder L.J., Aron D.C., Keith R.E., Kirsh S.R., Alexander J.A., Lowery J.C. (2009). Fostering Implementation of Health Services Research Findings into Practice: A Consolidated Framework for Advancing Implementation Science. Implement. Sci..

