# Supplementary material for: A Resilience-Based Nursing Intervention Framework for Internet Gaming Disorder in Saudi University Health Settings
Source: Healthcare (Basel). 2026 Aug 18;14(16):2599. doi: 10.3390/healthcare14162599 (PMC13512119; doi:10.3390/healthcare14162599)
Supplement: Supplementary file 1 [file healthcare-14-02599-s001.zip › healthcare-4478915-supplementary file S2.pdf]

Supplementary File S2: COREQ Checklist

Consolidated Criteria for Reporting Qualitative Research (COREQ)

Note: This is a framework development study following the Medical Research Council (MRC) guidance for complex interventions. It does not report a primary qualitative study per se. COREQ items are completed in relation to the framework development process, cultural consultation activities, and future qualitative work planned in Phase 1 (feasibility testing). N/A is used where items are not applicable to this study type. A semi:structured interview guide for future qualitative work is planned for the feasibility:testing phase but has not yet been drafted; no such appendix is included with this manuscript.

Domain / Topic	Item No.	Guide Questions/Description	Reported?
**Domain 1: Research Team & Reflexivity**	1	Personal characteristics: interviewer/facilitator (name, credentials, title)	**Yes**
	2	Credentials of the interviewer (e.g., PhD, MD)	**Yes**
	3	Occupation at time of the study	**Yes**
	4	Gender of the interviewer/facilitator	**N/A**
	5	Experience and training of the interviewer	**N/A**
	6	Relationship with participants: whether established prior to study commencement	**N/A**
	7	Participant knowledge of the interviewer: what participants knew about the researcher	**N/A**
	8	Interviewer characteristics: personal interests or assumptions, and theoretical framework	**Partial**
**Domain 2: Study Design**	9	Methodological orientation and theory: theory underlying the study (e.g., phenomenology, grounded theory)	**Yes**
	10	Sampling: purposive, snowball, theoretical, convenience	**Yes**
	11	Method of approach: how participants were approached (e.g., face:to:face, email)	**N/A**
	12	Sample size: number of participants in study	**Yes**
	13	Non:participation : any refusals or dropouts and reasons why	**N/A**
	14	Setting of data collection: location, context (e.g., home, clinic)	**Yes**
	15	Presence of non: participants: whether anyone else present during the interview	**N/A**
	16	Description of sample: key characteristics (e.g., demographics)	**Yes**
**Domain 3: Analysis & Findings**	17	Interview guide: questions, prompts, pilot testing	**N/A**
	18	Repeat interviews: whether repeated and if so, the number	**N/A**
	19	Audio/visual recording: whether interviews were audio or video recorded	**N/A**

Domain / Topic	Item No.	Guide Questions/Description	Reported?
	20	Field notes: whether field notes were made during or after the interview/focus group	**N/A**
	21	Duration: length of interviews or focus groups	**Yes**
	22	Data saturation: whether data saturation was discussed	**N/A**
	23	Transcripts returned: whether returned to participants for comment	**N/A**
	24	Number of data coders: how many researchers coded the data	**N/A**
	25	Description of the coding tree: whether authors provided a coding tree/thematic map	**N/A**
	26	Derivation of themes: whether themes were identified in advance or derived from the data	**Yes**
	27	Software: use of qualitative data software	**N/A**
	28	Participant checking: return of findings to participants for feedback/confirmation	**N/A**
	29	Quotations presented: to illustrate themes; identify quotes in terms of participant number	**N/A**
	30	Data and findings consistent: linkage between data and findings explicit	**Yes**
	31	Clarity of major themes: presentation of major themes	**Yes**
	32	Clarity of minor themes: description of diverse cases or discussion of minor themes	**Yes**
