# Supplementary material for: A Resilience-Based Nursing Intervention Framework for Internet Gaming Disorder in Saudi University Health Settings
Source: Healthcare (Basel). 2026 Aug 18;14(16):2599. doi: 10.3390/healthcare14162599 (PMC13512119; doi:10.3390/healthcare14162599)
Supplement: Supplementary file 1 [file healthcare-14-02599-s001.zip › healthcare-4478915-supplementary file S1.pdf]

Supplementary File S1. GUIDED Checklist

GUIDED: Guidance for Reporting Intervention Development Studies in Health Research. The entries below identify where each checklist item is addressed in the final revised manuscript.

Item	Checklist item	Manuscript response	Location
1	Context for development	Saudi university health services; Saudi sociocultural context, including gender-sensitive delivery and Islamic values integration.	Introduction; Section 3.4
2	Purpose of intervention development	To translate empirical findings on impulsivity, resilience, and IGD into a culturally adapted nursing intervention specification.	Introduction; Sections 2.1–2.2
3	Target population	Saudi university students reporting current video gaming engagement; proposed recipients are students presenting to university health services.	Sections 2.1; 3.1–3.3
4	Published development approach	Four iterative stages informed by Medical Research Council guidance; Behaviour Change Wheel/Intervention Mapping; Bernal's Ecological Validity Framework.	Sections 2.2–2.4
5	Evidence from different sources	Companion empirical dataset; systematic reviews of gaming-disorder and resilience interventions; published regional literature; nursing-professional consultation.	Sections 1; 2.1–2.2
6	Use of theory	I-PACE, Dual-Process Theory, Stress-and-Coping model, and COM-B analysis informed targets and intervention functions.	Sections 2.2–2.3
7	Components from an existing intervention	No named intervention programme was reproduced. Evidence-	Sections 2.2; 3.3

		based intervention elements were adapted at the level of behaviour-change techniques and therapeutic approaches.	
8	Guiding principles and prioritised factors	Cultural appropriateness, non-stigmatising access, clinical safety, tiered intensity, student autonomy, optional family involvement, and gender-sensitive delivery.	Sections 3.1–3.4
9	Stakeholder contribution	Saudi mental-health nursing professionals informed drafting. No direct student stakeholder involvement occurred during this phase; this is identified as a limitation and future priority.	Section 2.2; Section 4.5
10	Changes during development	The earlier proposal for sex-differentiated content emphasis was not retained because the available evidence did not establish different treatment needs. Remaining content is common across groups, with future adaptations to be co-designed.	Section 3.4
11	Changes for subgroups	Single-sex group delivery and optional, student-led family communication are specified. Other tailoring requires participatory testing.	Section 3.4
12	Uncertainties at end of development	Screening validation, acceptability, safety, delivery feasibility, workforce capacity, fidelity, and clinical outcomes remain untested.	Sections 3.5; 4.5; 5
13	Use of TIDieR guidance	The intervention is described using the TIDieR checklist; a completed checklist is provided as Supplementary File S2.	Section 2.2; Supplementary File S2

14	Open-access reporting	The manuscript is prepared for submission to Healthcare, an open-access journal.	Title page and journal submission files

Reference: Duncan, E.; O'Cathain, A.; Rousseau, N.; et al. Guidance for Reporting Intervention Development Studies in Health Research (GUIDED): An Evidence-Based Consensus Study. BMJ Open 2020, 10, e033516. https://doi.org/10.1136/bmjopen-2019-033516.
